# ACTH-secreting pituitary carcinoma with TP53, NF1, ATRX and PTEN mutations Case report and review of the literature

**DOI:** 10.1007/s12020-021-02954-0

**Published:** 2022-02-16

**Authors:** Piotr Sumislawski, Roman Rotermund, Silke Klose, Anne Lautenbach, Annika K. Wefers, Celina Soltwedel, Behnam Mohammadi, Frank Jacobsen, Christian Mawrin, Jörg Flitsch, Wolfgang Saeger

**Affiliations:** 1 Department of Neurosurgery, University Medical Center Hamburg-Eppendorf, Martinistr.52, 20246 Hamburg, Germany; 2 Department of Internal Medicine/Endocrinology, Otto von Guericke Universität Magdeburg, Magdeburg, Germany; 3 III. Department of Medicine, University Medical Center Hamburg-Eppendorf, Hamburg, Germany; 4 Institute of Neuropathology, University Medical Center Hamburg-Eppendorf, Hamburg, Germany; 5 Mildred Scheel Cancer Career Center HaTriCS4, University Medical Center Hamburg-Eppendorf, Hamburg, Germany; 6 Institute of Pathology, University Medical Center Hamburg-Eppendorf, Martinistr, Hamburg, Germany; 7 Institute of Neuropathology, University of Magdeburg, Magdeburg, Germany

Pituitary carcinoma (PC) is a very rare tumor entity of the sella turcica, representing 0.1–0.5% of all PitNETs tumors [1–5]. Based on the WHO Classification, it is defined as pituitary tumor with confirmed craniospinal and/or systemic metastases [6]. Most of them present CNS only (45,2%) or extra CNS (38,7%) metastases. Synchronous extra- and CNS metastases are less common (16,1%) [7]. It is not known whether the tumors develop predominantly from PitNETs after a longer clinical course or de novo [8]. PCs can be hormonally inactive or active (ACTH-, PRL-, GH-, TSH- FSH-, LH-secreting), but far most of them are ACTH- or PRL-secreting tumors [8, 9]. There is little known about the genetic background of this tumor entity, because most of the information comes from case reports and singular larger case series. *ATRX* [10, 11]*, CDKN2A* [11]*, CDKN2B* [11]*, SDHB* [12], *TP53* [11, 13] mutations have been identified in primary [13] and *ATRX* [10, 11]*, CDKN2A* [11]*, CDKN2B* [11]*, H-Ras* [14] mutations in metastatic tumors. *MSH2* germline mutation was described in one case report [15]. *PTEN* mutations were reported without localization [16]. The tumors have a poor prognosis with a 66% mortality rate after 1 year and up to 80% after 8 years [1, 17].

Treatment options include surgery, chemotherapy, hormonal therapy, and/or radiotherapy. There are many different chemotherapeutic protocols. To the most commonly used chemotherapeutics include temozolomide, CCNU + 5-fluoruracil. Especially in ACTH-secreting PCs other agents, which lower ACTH and cortisol secretion, are additionally used. As most ACTH-secreting tumors express somatostatin receptor type 5, pasireotide as potent somatostatin analog with high affinity to somatostatine receptor type 5 showed significant suppression of ACTH and cortisol secretion [18]. Mitotane and ketoconazole as steroidogenesis inhibitors can support the treatment by reducing the cortisol levels and present an alternative to bilateral adrenalectomy [10, 19, 20].

In this paper, we present the case of an ACTH-secreting PC with liver and thoracic vertebrae metastases. For further characterization, DNA from the primary tumor and liver metastases were isolated. DNA sequencing revealed *TP53*, *NF1* mutations in the primary tumor, and *TP53, NF1, PTEN,* and *ATRX* mutations in liver metastases. Based on our results and the literature, we discuss the genetic origin of PC and the molecular principles of their metastases (Fig. 1).

**Fig. 1:**
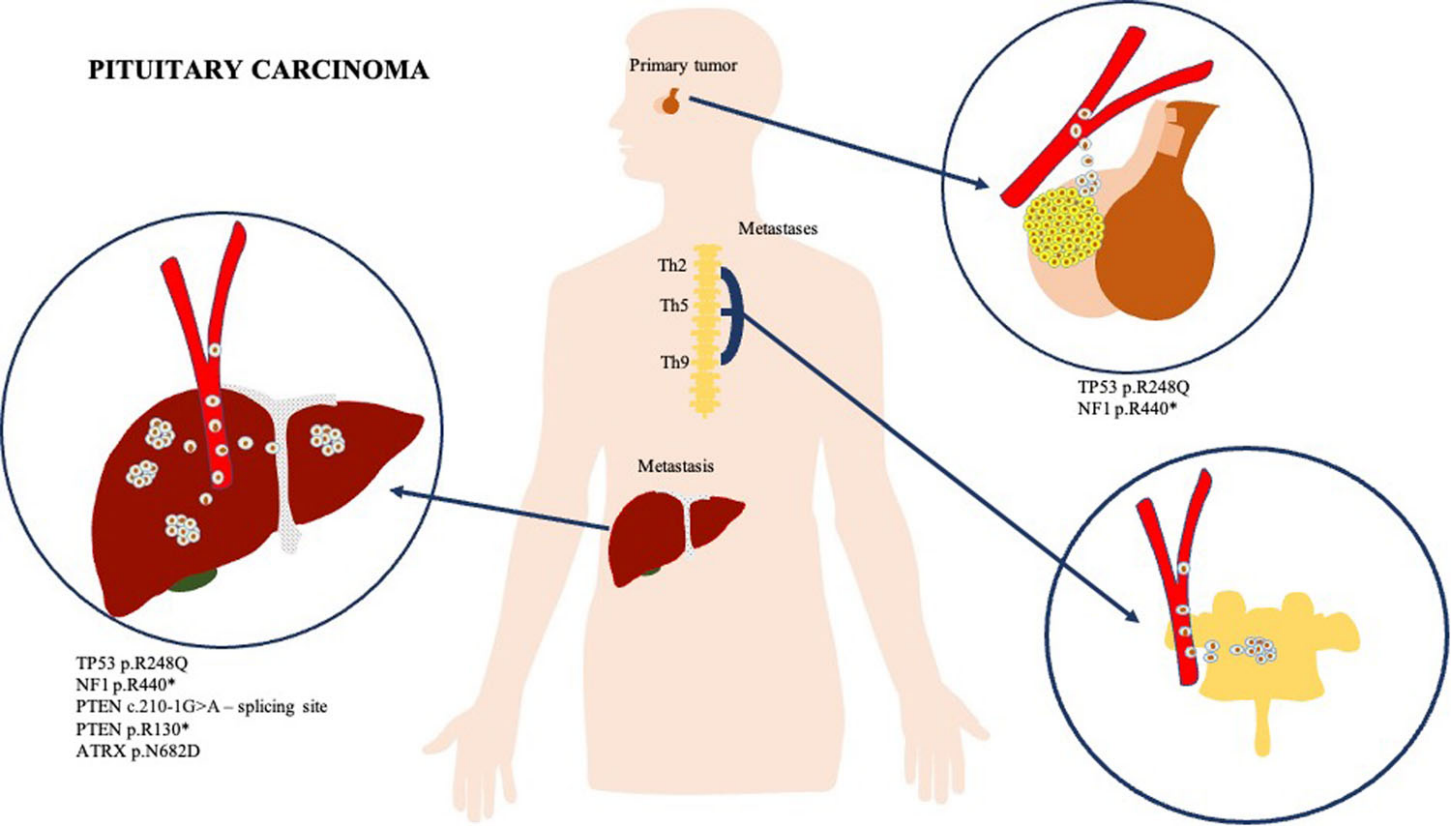
Graphical abstract

## Case report

**Fig. 2:**
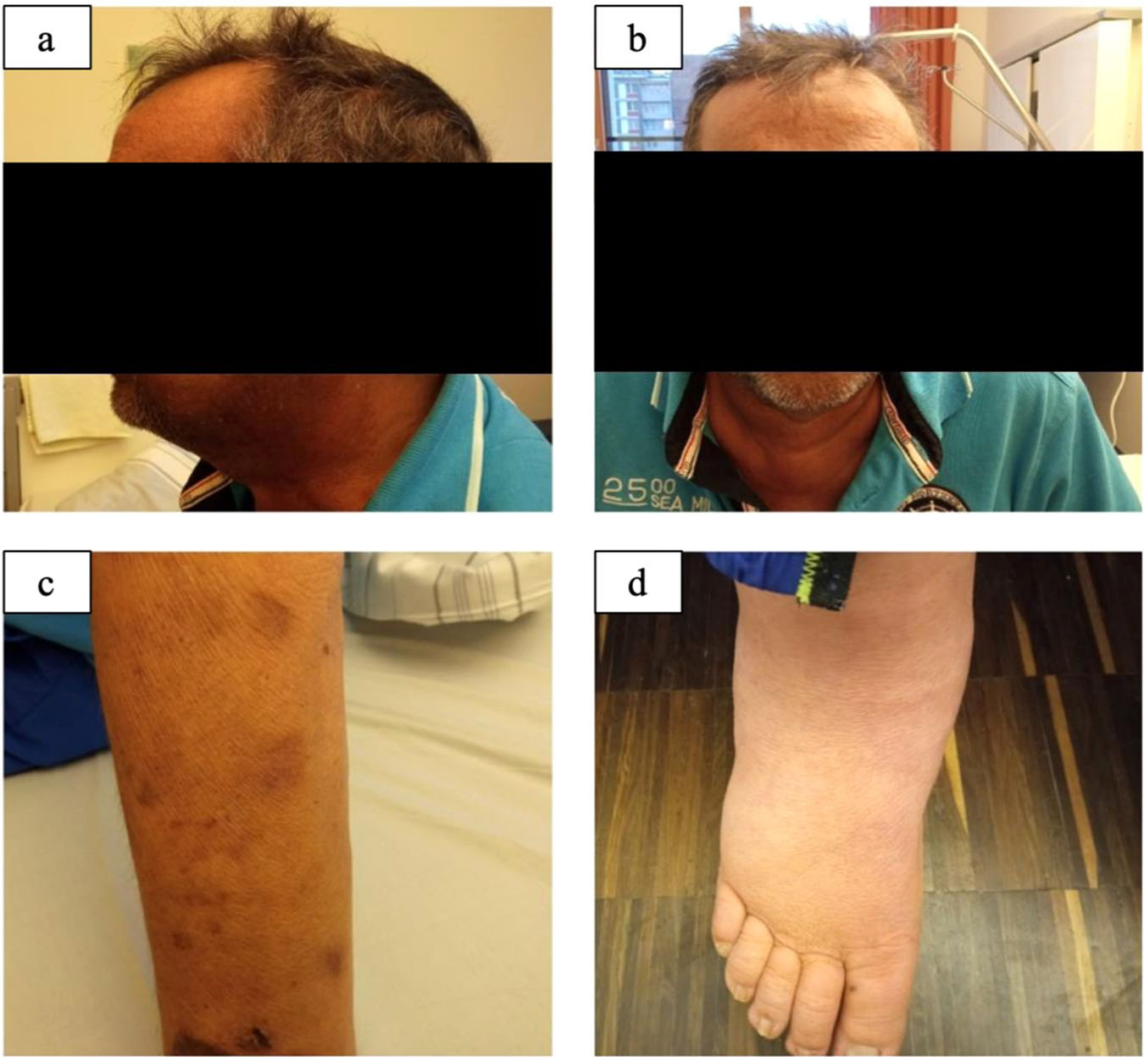
Clinical examination. Hyperpigmentation of the skin predominantly in the face (**a**, **b**) and at upper extremity (**c**) in comparison to lower extremity (**d**)

**Fig. 3:**
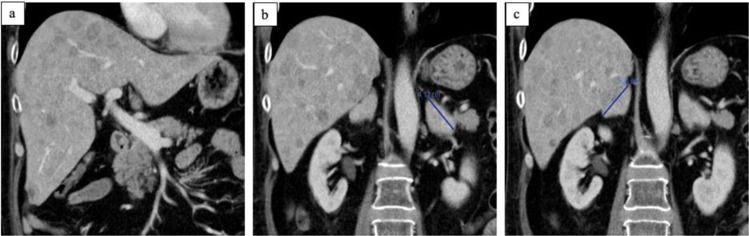
Liver metastases. Liver metastasis (**a**−**c**) with massively enlarged adrenal glands (**b**, **c**)

**Fig. 4:**
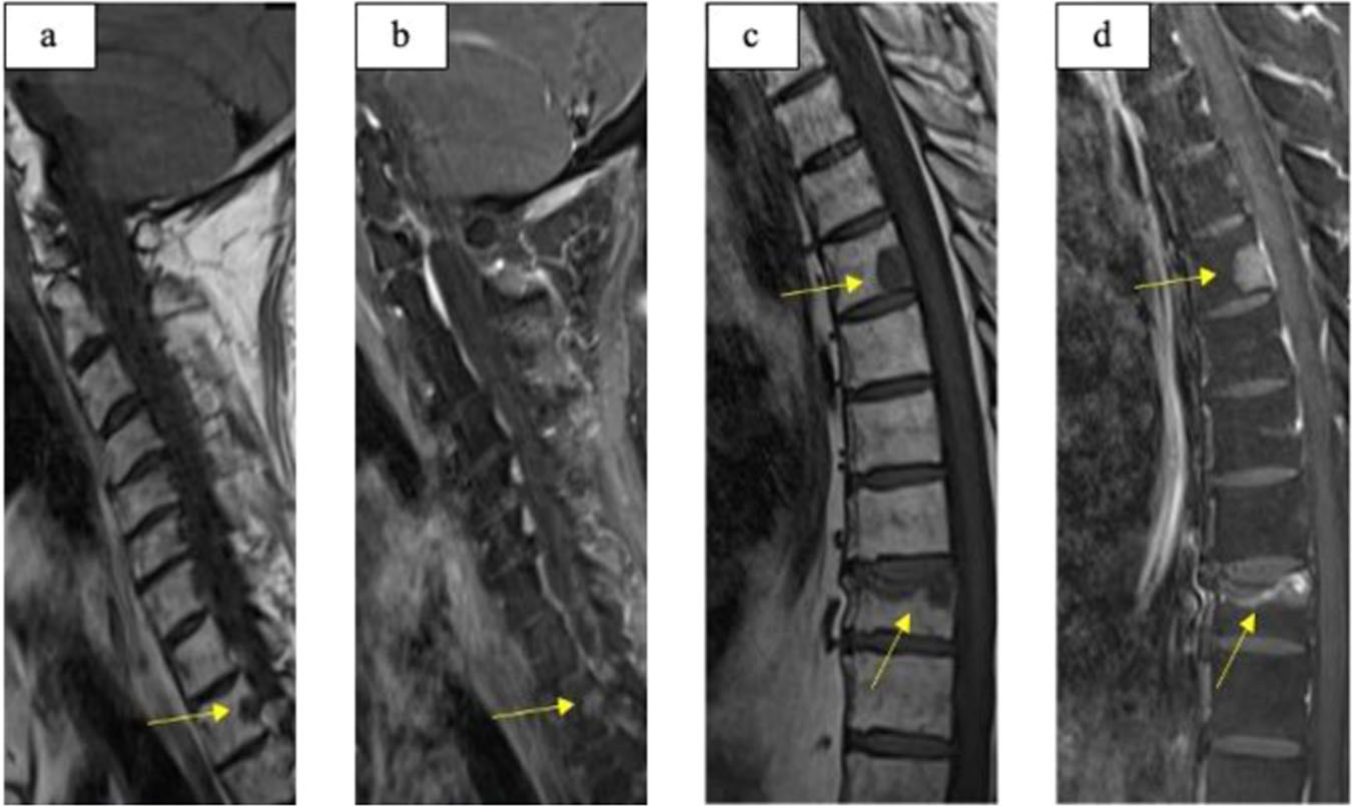
Vertebral metastases. Metastases in the vertebral bodies of Th2 (arrows) -T1 seq (**a**), T1 seq with contrast agent (**b**) and Th5, Th9 (arrows) -T1 seq (**c**), T1 seq with contrast agent (**d**)

A 53-years old male was referred for repeated surgery of a persisting pituitary neuroendocrine tumor (PitNET) with a 2-year history of arterial hypertension and diabetes mellitus, clinical (Fig. 2), and laboratory signs (Table 1) of Cushing’s disease, hypopituitarism and hypokalemia. Eight months before admission, the patient had been operated at another neurosurgical center with initial diagnosis of ACTH-secreting PitNET with strongly increased proliferation. After operation, the patient received adjuvant radiation (54 Gy) and systemic therapy with metyrapone (3 g) and ketoconazole (400 mg) for persisting hypercortisolism. In the initial laboratory testing, a relevant decline in cortisol and ACTH levels was seen, requiring temporary hydrocortisone replacement. Control testings after 4 months revealed tumor relapse. The ophthalmologic examination before the second surgery was unremarkable. An elective exoscopic transsphenoidal surgery was performed. Postoperatively, the patient remained neurologically intact without any signs of liquorrhea, headaches, nausea, or emesis. Laboratory testing showed further pituitary insufficiency, hypokalemia under potassium substitution, no significant improvement of cortisol and ACTH levels. There were no signs of diabetes insipidus or SIADH. Because of persistent, strongly increased ACTH and cortisol levels, a thoracic and abdominal CT and a craniospinal MRI were performed to search for ectopic sources of ACTH. They revealed multiple lesions suspicious for metastases in the liver (Fig. 3) and in the body of the thoracic vertebrae 2, 5, and 9 (Fig. 4). Sella MRI revealed normal postoperative finding without any residual tumor. The adrenal glands were massively enlarged as a result of ACTH stimulation (Fig. 3). The hepatic laboratory tests revealed elevated transaminases (AST and ALT), ALP, and especially GGT, as a sign of liver dysfunction which remained stable under treatment with ketoconazole (Table 2). A CT-guided biopsy was performed for histological assessment. Pathologic examination of the liver revealed many small nests of tumor cells, compatible with PC metastasis.

## Pathohistology of liver metastases

**Fig. 5:**
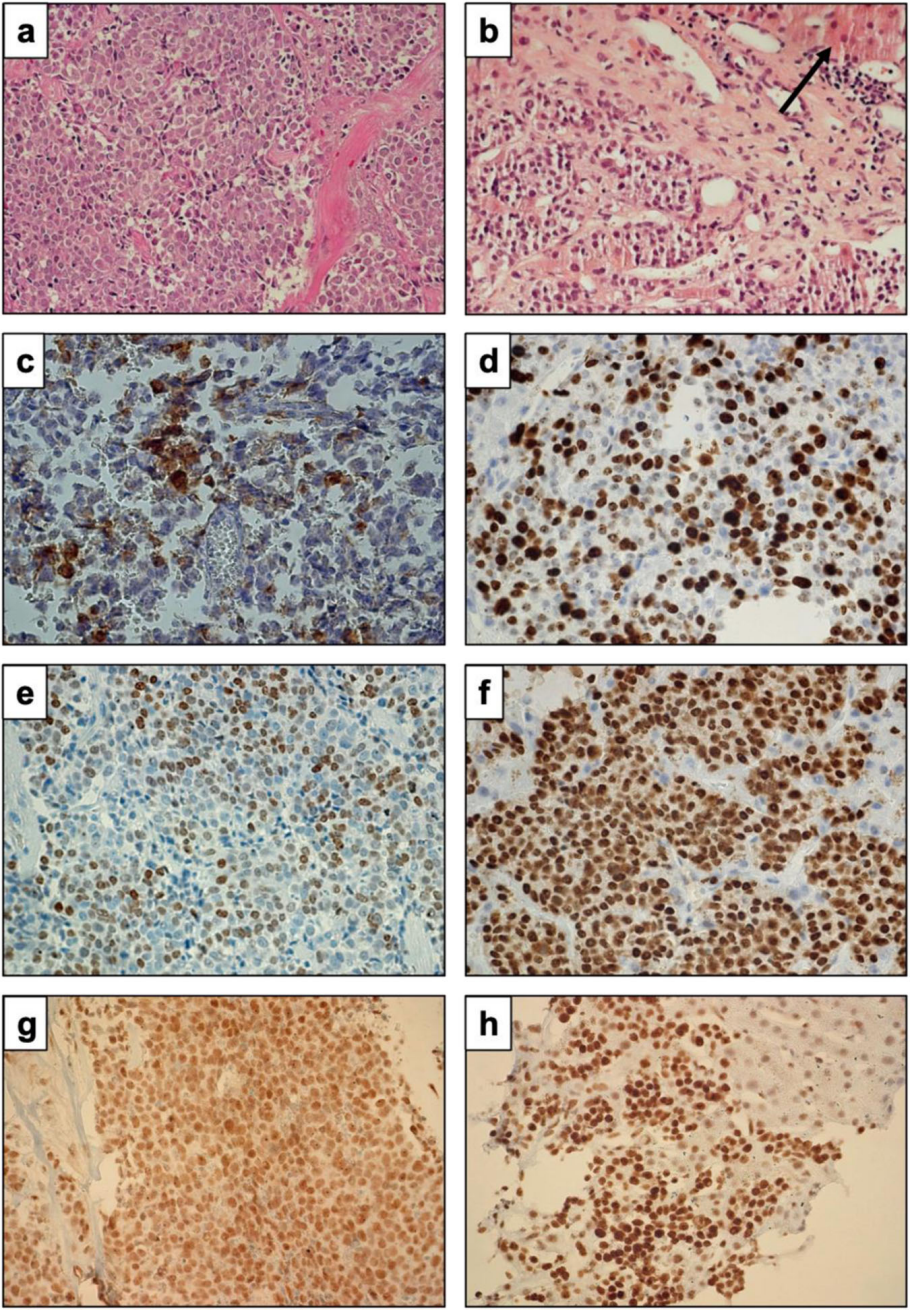
Histological examination. **a** Pituitary tumor: HE, 250×, **b** liver metastasis: HE (arrow: liver tissue) 440×, **c** pituitary tumor: ACTH expression in 20% of tumor cells, 440×, **d** metastasis: Ki-67 index 50–60%, 440×, **e** pituitary tumor T- pit expression in nuclei, 250×, **f** metastasis: p53 expression in all nuclei, 440×, **g** pituitary tumor: ATRX expression retained, 250×, **h** metastasis: ATRX expression retained (right upper corner-l liver tissue), 440×

Biopsy shows very small foci within the blood sinus and one larger focus of tumor tissue composed of densely arranged small to medium-sized cells with chromatin-rich nuclei and poor cytoplasm. Manually counted mitoses had a median value of 20 /10 HPF. Immunostains for ACTH were positive, but the transcription factor for pituitary ACTH cells (T-pit) was not expressed. Ki-67 index was very high (60%) (Fig. 5d). p53 was expressed in nearly all tumor cell nuclei (Fig. 5f) (Table 3). ATRX expression was retained (Fig. 5h).

## Pathohistology of pituitary tumor

The structure and immunostains of the pituitary tumor were very similar to the tumor in the liver. Therefore, we can clearly state that either the tumor in the pituitary and in the liver were metastases of a somewhere else localized tumor or the pituitary tumor was the primary. This question could be answered by the expression of T-pit (Fig. 5e) in the pituitary tumor since this transcription factor is the lineage marker for the pituitary ACTH cells (Fig. 5c). ATRX expression was retained (Fig. 5g). EGFRVIII was not expressed.

## Next-generation-sequencing

DNA panel sequencing of the sellar and hepatic tumors revealed the same mutations of *TP53* (NM_0005465:c.743G > A, p.R248Q) and *NF1* (NM_001042492.2:c.1318C > T, p.R440*) in both tumors. Additionally, we found two *PTEN* mutations (NM_000314.6:c.388C > T (p.R130*) and c.210-1G > A (splice site) as well as an *ATRX* mutation(NM_000489.4:c.2044A > G, p.N682D) in the liver tumor only. This confirmed that the pituitary tumor was the primary while the liver tumors were metastases.

## Sanger-sequencing

USP8 exon 14 sequencing along with and USP48 region encompassing hotspot in USP domain revealed no mutations or deletions.

Because of multiple disseminated liver and vertebral metastases, the palliative treatment with temozolomide, mitotane, and pasireotide was started. The patient died 10 months after the first diagnosis of the pituitary tumor.

**Table 1:** Pre- and postoperative laboratory results with reference ranges

Test	Result preoperative	Results 3rd postoperative day	Reference range
ACTH	3534	3360	~ 46 ng/l
Cortisol	1621	1835	52,7–224 µg/l
GH	<0.15	<0.15	~ 16 mU/l
IGF-1	55.6	50.3	48–209 µg/l
TSH	0,04	0.04	0.55–4.76 mU/l
fT4	16.3	11.5	11.5–22.7 pmol/l
fT3	3.0	2.1	4.5–6.5 pmol/l
FSH	0.6	0.5	1.4–18.1 U/l
LH	<0.07	<0.07	1.5–9.3 U/l
Testosterone	3.87	3.13	0.86–7.88 µg/l
Prolactin	2.1	1.8	2.1–17.7 µg/l
Potassium	2.5	3.2	3.5–4.6 mmol/l
Sodium	136	137	135–145 mmol/l

**Table 2:** Liver function laboratory results with reference ranges

Test	Results	Reference range
AST	109	<50 U/l
ALT	146	<50 U/l
GGT	12888	<73 U/l
ALP	223	46–116 U/l

**Table 3:** A summary of the staining

Tissue	ACTH	Ki-67	Mitoses per 10 HPF	p53	T-Pit	synaptophysin	chromogranin
Pituitary	Strong	60%	20	~100%	Positive	Strongly positive	Negative
Liver	Strong	60%	20	~100%	Negative	Strongly positive	Strongly positive

**Table 4:** Characteristics of studies included in this review

Pat. No.	Gene	CDS mutation	AA mutation	Germline(G)/primary tumor (PT)/metastasis(M)	Authors & year
1	ATRX	c.134_6217del	p.D45-K2027del	PT	Casar-Borota et al. 2021
2		c.748C > T	p.Arg250Ter	PT	
3		c.6679delG c.3583delA	p.Asp2227fs p.Arg1195fs	PT	
4		c.4048_4049delGG c.6661G > T	p.Gly1350fs p.Glu2221Ter	PT	
4		c.4048_4049delGG	p.Gly1350fs	M	
5		c.595_6699del	p.N199-K2233del	PT and M	
6		Deletion of exon 3 to 27	No data	PT and M	Casar-Borota et al. 2017
7		No data	No data	Not mentioned	Guo et al. 2018
5	CDKN2A	c.1_501del	p.M1-A167del	PT and M	Casar-Borota et al. 2021
5	CDKN2B	c.1_414del	p.M1-D138del	PT and M	
8	H-Ras	c.34G > C	p.G12R	M	Pei et al. 1994
9		c.52G > A	p.A18T	M	
10		Codon 3 del	-	M	
11	MSH2	c.1587delA	p.E530Kfs	G	Bengtsson et. al. 2007
7	PTEN	No data	No data	Not mentioned	Guo et al. 2018
12	SDHB	c.587G > A	p.Cys196Try	PT	Tufton et al. 2017
2	TP53	c.524G > A	p.Arg175His	PT	Casar-Borota et al. 2021
4		c.644G > A	p.Ser215Asn	PT	
7		No data	No data	Not mentioned	Guo et al. 2018
13		c.742C > G	p.R248G	PT	Tanizaki et al. 2007
14		c.404G > T	p.C135F	PT	

*CDS mutation* coding DNA sequence mutation, *AA mutation* amino acid mutation.

### Review of literature for mutation analyses

A literature review via PubMed using the search terms ‘pituitary carcinoma’, ‘pituitary carcinomas’ combined with ‘mutation’ was performed. Only studies with a confirmed diagnosis of PC and information about the mutational status were included. Total number of 7 studies containing 34 patients from which only 14 revealed pathologic gene variants via gene sequencing. *TP53* mutations were identified in the primary tumors [11, 13] (Table 4). Loss-of-function mutations of *ATRX* gene were described both in primary and metastatic PC [8, 11, 16] (Tab.4). Higher incidence of *ATRX* mutations among recurrent comparing to primary PitNETs may indicate a possible contribution to tumor progression [21]. One patient with PC harboring a loss-of-function *SDHB* mutation in and history of paraganglioma was described [12] (Table 4). Ras gene analysis involving K-, H- and N-Ras revealed 2 different *H-Ras* mutations in PC metastasis [14] (Table 4). A patient with Lynch Syndrome and germline *MSH2* mutation in a PC was reported [15] (Table 4).

## Discussion

*TP53*, *NF1*, and *PTEN* are well known tumor suppressor genes. *P53* and *NF1* are involved in Ras-activity regulation. p53 suppressor effect on RAS-activity is mediated by BTG2 [22, 23]. Several different domains are responsible for apoptosis, growth repression or DNA repair [24–26]. This protein is also involved in other cellular functions such as control of cell cycle through p21 and is self-controlled by MDM2 protein. As *TP53* mutations were described by primary tumors, they may be involved especially in the PC tumorigenesis. *TP53* p.R248Q. mutation is localized in the DNA-binding domain (Fig. 4) and its effect can be mediated by both BTG2 and NF-κB [22].

NF1 on the other hand negatively regulates RAS pathway by inducing dephosphorylation of Ras-GTP to Ras-GDP [27]. This protein is made from several domains with different functions [28, 29]. *NF1* p.R440*, a nonsense mutation, causes protein-truncating variant without essential domains such as Cysteine/Serine-rich domain or GTPase-activation protein-related domain (Fig. 5).

PTEN protein is built up of different domains including phosphatase domain [30, 31], which is responsible for converting PI (3,4,5) P_3_ to PI (4,5) P_2_ and thus antagonizing the PI3K pathway ^32^. Both *PTEN* c.210-1 G > A and p.R130* mutations (Fig. 4) cause loss-of-function which can consequently activate PI3K pathway. It could then promote tumor metastases by inducing epithelial-to-mesenchymal-transition and cytoskeletal remodeling, which can at the end increase the tumor motility [33, 34]. PTEN loss or its low expression was correlated with a higher risk of metastasis [35]. PTEN mutation however described by Guo et al. by PC, lacked basic information regarding the origin of examined sample and the localization of detected mutation [16].

ATRX is a transcriptional regulator and its mutations including loss-of-function were detected in PC [8, 11, 16]. ATRX loss-of-function may induce telomere instability and promote alternative lengthening of telomeres (ALT), as ATRX maintains their structure and function by interacting with DAXX and histone H3.3 variant [8].

Tumor cells harboring of *ATRX* p.N682D mutation in metastasis retained ATRX expression in immunostains (Fig. 4h), which can suggest the preserved function of ATRX protein.

*USP8* and *USP48* gene mutations are frequent in corticotroph PitNETs [36, 37]. Gene sequencing of USP8 and *USP48* in primary tumor revealed no mutations or deletions in hotspot regions. Sbiera et al. described all *USP48* mutated cases only with *TP53* wildtype variant indicating that they can be mutually exclusive [37].

ATRX, p53, Ki67 immunostains may be useful in the early diagnostic of PC. Loss of ATRX expression may indicate PC in immunochemistry, as around 20% of PC harbor loss-of-function mutations of this gene. On the other hand, increased Ki67 and p53 expression over the cut-off values Ki67 (≥4%) and p53 (≥2%) suggest aggressive PiNETs and with higher values even PC [38].

**Fig. 6:**
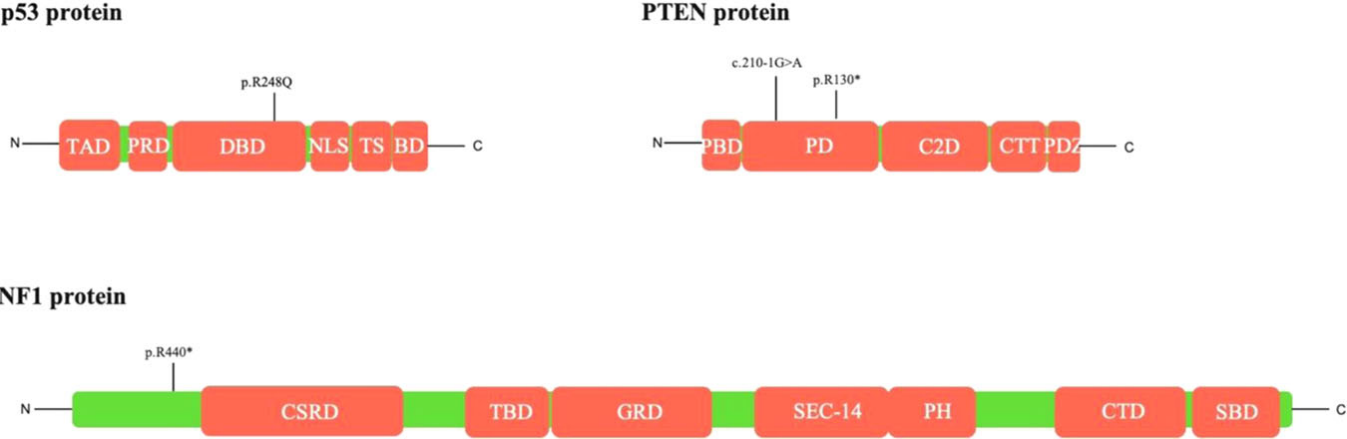
P53, PTEN, and NF1 protein structure. p53, PTEN, and NF1 protein structure from N-Terminal to C-Terminal with localization of detected mutations. TAD- transcriptional activation domain, PRD- proline-rich domain, DBD- DNA-binding domain, NLS- nuclear localization signal, TS- tetramerization domain, BD- basic domain, PBD- PIP2 binding domain, PD-phosphatase domain, C2D- C2 domain, CTT- C-terminal tail, PDZ-PSD95/Disc large/Zonula occludens-1 domain, CSRD-Cysteine/Serine-rich domain, TBD- tubulin-binding domain, GRD- GTPase-activation protein-related domain, SEC-14- SEC-14 domain, PH- pleckstrin homology domain, CTD-Carboxy terminal domain, SBD-Syndecan-binding domain

**Fig. 7:**
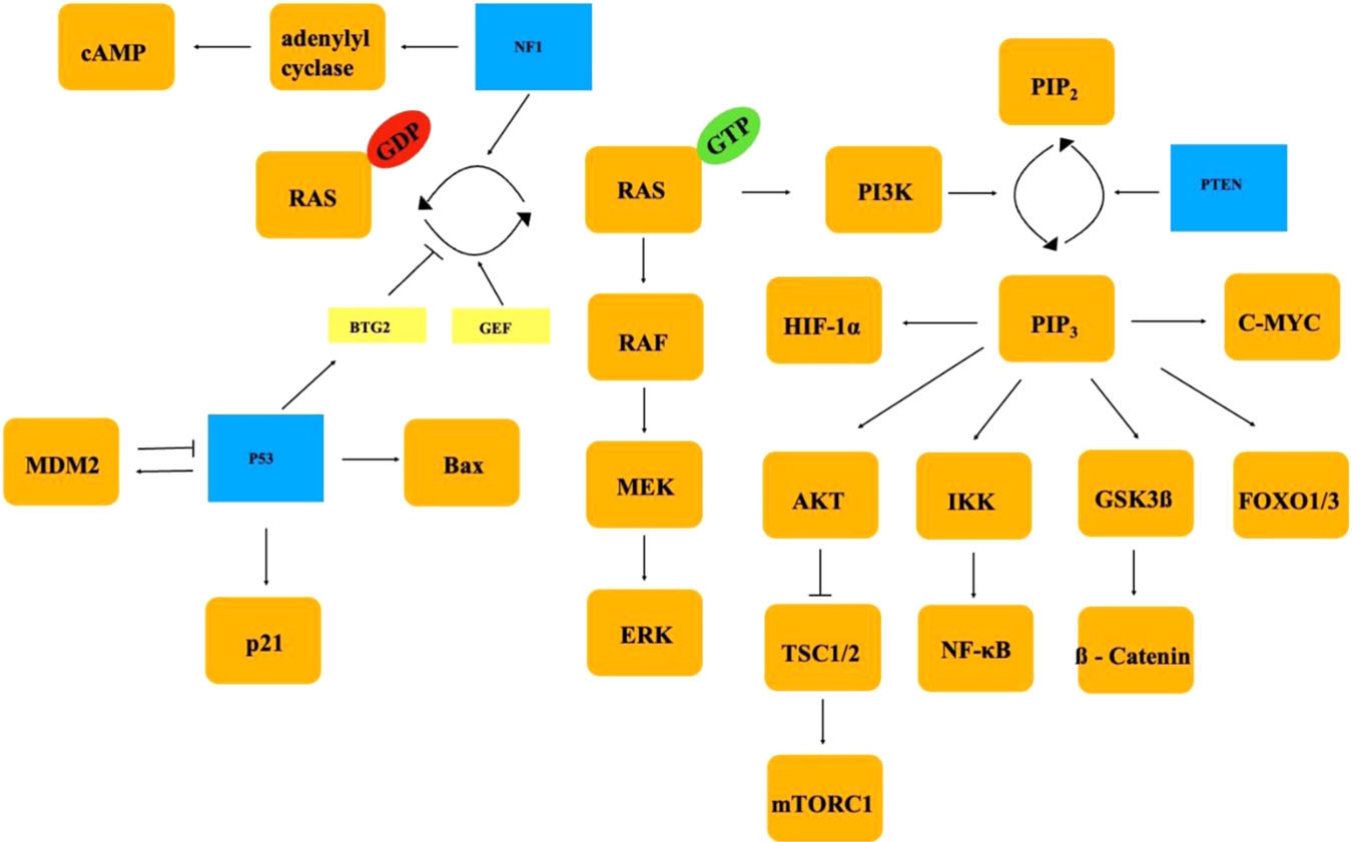
Signaling networks regulated by NF1, P53 and PTEN proteins. Schematic presentation of signaling network with pathways regulated by NF1, P53, and PTEN proteins

*NF1*, *TP53*, and *PTEN* mutations lead to activation of several well-known signaling pathways (RAS, RAF, MAPK, ERK, PI3K, Akt) [22–35] (Fig. 6). From a therapeutic view, they could offer a potential goal for targeted drug therapy, as RAF-, MEK-, ERK-, PI3K- and Akt- inhibitors have been successfully tested in many clinical trials [39–41]. In case of TP53 mutation novel therapeutic agents such as APR-246 converting mutant to wild type p53 or bispecific antibody against mutant p53 could be implemented in the oncological therapy [42]. For tumors with ATRX loss-of-function and following ALT process and impaired DNA repair, an epigenetic therapy applied by G-quadruplex-interacting compounds may be effective, as could restore genomic stability [43] (Fig. 7). Clinical studies including immune checkpoint inhibitors (pembrolizumab, ipilimumab, or nivolumab) by aggressive PiNETs along with PCs showed ambiguous results, as one patient exhibited tumor regression and another faced with tumor progression after the treatment [44]. It confirms that PCs are heterogenous group of tumors and require more complex and personalized diagnostic approach to identify the genetic drivers and to try to establish molecularly targeted therapy in the future.

## Conclusion

Pituitary carcinoma(PC) is a devastating disease with high mortality rate. The molecular background for the development of this rare tumor entity and the mechanism of metastasis are unknown. There are only a few case reports and singular larger case series considering only restricted number of sequenced genes by the PC [8, 11–16]. From our case report and review of the literature we propose special improvements of diagnostic approach in case of PC suspicion by application of NGS for at least several genes found in PC biology ATRX, CDKN2A, CDKN2B, H-Ras, NF1, PTEN, SDHB, MSH2, and TP53 genes, as well as these involved in PitNET biology e.g., USP8 or USP48. This technique may be implemented into clinical practice to detect the genetic drivers for planning targeted therapies for PC as several identified gene mutations are potential targets for pharmacological therapy.

## Methods

### Microscopy and immunochemistry

Tumor tissue was fixed in 4% paraformaldehyde directly after surgical resection, dehydrated, embedded in paraffin, and then sectioned at 2 µm according to standard lab protocols. For all immunohistochemical stains paraffin-embedded tissue was deparaffinized, rehydrated. All immunohistochemical stainings were performed using automatic staining machines (Ventana BenchMark TX and Ventana Discovery Ultra), Roche Diagnostics, Mannheim, Germany). The following primary antibodies were used: ATRX(1:400, HPA001906, Atlas Antibodies, Bromma, Sweden), GH(1:1000, PA0704, Leica Biosystems, Buffalo Grove, IL, United States), Prolactin(1:1000, ab11301, Abcam, Cambridge, UK), TSH(1:10000, Epredia, Portsmouth, NH, United States), ACTH(1:500, RP045, Diagnostic BioSystems, Hanhgzhou, China), FSH(1:200, M3504, DAKO, Glostrup, Denmark), LH(1:300, M3502, DAKO, Glostrup, Denmark), Pit-1(1:200, HPA041646, Sigma-Aldrich, Taufkirchen, Germany), T-pit(1:1500, AMAb91409, Atlas Antibodies, Bromma, Sweden), TTF-1(1:50, M3575, DAKO, Glostrup, Denmark, Ki-67(1:750, 275R-15, Cell Marque, Rocklin, CA, United States), p53(1:800, M7001, DAKO, Glostrup, Denmark), chromogranin(1:800, M0869, DAKO, Glostrup, Denmark, synaptophysin (1:500, M7315, DAKO, Glostrup, Denmark), EGFRvIII (1:250, T170B620, Absolute Antibody, Oxford, Great Britain).

### DNA isolation

The tissue was further sectioned 10 times at 10 µm according to standard lab protocol. HE stains were utilized for the selection of tumor area. Tumor tissue was then manually microdissected using a fine needle under an inverted microscope. The DNA was isolated using Maxwell^®^ RSC DNA FFPE Kit (AS1450, Promega).

### Next-generation-sequencing

DNA panel sequencing was done using a self-customized targeted panel, manufactured by Qiagen (CDHS-21330Z-424). This panel targets the complete coding regions and splice-sites of six genes (ATRX, EGFR, NF1, NF2, PTEN, TP53), as well as mutation hotspots of further 14 genes (AKT, BRAF, CTNNB1, FGFR1, FGFR2, H3F3A, HIST1H3B, HIST1H3C, IDH1, IDH2, KRAS, PI3CA, PIK3R1, TERT-promoter). The library was constructed according to the manufacturer’s instructions. Sequencing was done on an Illumina MiniSeq sequencing system (paired-end, 2 × 151 bp, average coverage 500x). Data were analyzed with the Qiagen CLC Genomics workbench, using a self-customized workflow. Variants were annotated with information from the 1000 genome project, dbSNP, ClinVar and COSMIC. Only variants with an allele frequency ≥ 5% and a total target coverage of ≥40x were analyzed further. Variants not annotated by ClinVar were additionally analyzed with VarSome (www.varsome.com).

### Sanger sequencing

Primers previously described by Sbiera et al. were used for both amplification and sequencing the specific regions of USP8 and USP48 genes [37]. A PCR reaction volume of 25 μl containing 40 ng of template DNA, 0.1 μM of each primer, 100 μM dNTPs (deoxyribonucleotide triphosphates), Dream Taq polymerase buffer, and 1.25 U Dream Taq DNA Polymerase was prepared and amplified after initial denaturation at 95 °C for 3 min followed by 35 cycles of denaturation at 95 °C for 30 s, annealing at 55 °C for 45 s, and elongation at 72 °C for 60 s. PCR products were sequenced using Mix2Seq Kit NightXpress and performed by Eurofinsgenomics.
